# Suicide

**Published:** 1876-01

**Authors:** 


### SUICIDE.

THE most efficient preventive of this crime is a strong reprobative public sentiment. It is regarded with a kind of horror. There is an instinctive drawing back from the self-murderer on the part of most people, as if there was no wish to have anything to do with a character around which there gathers at once a discreditable mystery. Almost the first inquiry on hearing of a suicide is, *what evil has he done?* It seems to be a general feeling that it has some connection with remorse, which, in its turn, is suggestive of crime. And so intense is the unexpressed sentiment, that suicide has been committed to cover some crime, or to escape the imputation of wrong; the very first effort on the part of friends, is to promote an impression that it was the result of insanity.

We unhesitatingly assert, that the palliation of suicide is a wrong done to society and to good morals. Let it be a deep, a general, an abiding sentiment, that suicide and criminality are inseparable, and an important step will have at once been taken towards its prevention.

Some years ago, this species of depravity became so prevalent among the women, the authorities decreed that the body of every such person found should be hung naked at the main entrance of the town. The effect was an instantaneous cessation of the unnatural act.

We do not undertake to say that every suicide is a legal criminal, but we do say unhesitatingly, that a suicide is a coward and a fool. A coward, because he runs from trouble; a fool, because he rushes into the presence of his Maker with a dagger in his hand and a sin in his heart.

The papers of the country, secular and religious, have made a great ado lately, about the suicide of an unmarried man, aged thirty-five, of an irreproachable private character, up to his last act; a man of genius and of education. He was an enthusiastic "Spiritualist," and the use made of the fact is, "*Spiritualism is*

*a pernicious delusion.*" And we almost grit our teeth in the impatience which attends the expression of the sentiment. Fair play is a jewel the world over. Let us have it here. If every creed is to be voted false, wicked and dangerous, because an advocate of it has been driven to suicide, then every creed under the sun, and every great pursuit, is false, wicked, and dangerous, our Holy Religion not excepted. The wonder to our mind is, that any man, with even moderate pretensions to a logical mind, would allow himself to employ such a baseless argument. There is not one of us that would not regard with contempt the man who would urge as a reason, religion was a delusion, because persons are every now and then precipitating themselves into eternity as the effect of "*strong religious excitement,*" as it is benevolently termed.

As stated in our December number, an eminent divine seeks affectionately to cloak over the self-destruction of a talented brother, by the argument that his mind was unbalanced by intense devotion to some particular branch of study, his general health being at the same time much impaired; and the argument is given, with an editorial backing, in the columns of one of the oldest, most widely circulated and ably edited religious newspapers in the United States; and what is more wonderful still, without rebuke, as far as we have seen, by a single christian editor in the land. But when a "Spiritualist" commits suicide, scarcely a paper fails to blazon it abroad with various exclamatory marks and startling phrases—*Horrible effects of Spiritualism !!!* and the like.

Now compare the two cases: both were unmarried, of about the same age; both educated; both possessing mental qualities entitling to the name of "*genius;*" and both, too, labored under bodily disease of an aggravated character.

The view which we take of these cases, and hence their introduction here, is, that the crime of the self-destroyer is not chargeable logically, either to religion, to scientific research, or to spiritualism, but is chargeable to the sin of being in wretched ill-health. No man, ordinarily, in the exercise of a moderate degree of intelligence as to the physiological laws of his body, can labor under wretched ill-health, except from unavoidable occurrences.

In the progressive intelligence of the age, we must march up to the truth, and face it manfully , and more, we must consistently and conscientiously live up to its dictates, physically as well as morally, inasmuch as the same Great Mind which ordained the moral law has enacted the physiological, and no millennium can ever dawn on our eyes, until both are lovingly obeyed.

Medical men of all schools agree that the mass of suicides are the result of bodily infirmity of some kind. Perhaps, in as large a number of cases as three out of four, the result is brought about as follows. There is too much blood in the body, and as is always the case, under such circumstances, it is "bad blood;" bad for several reasons—it is imperfect, impure, and too thick, to flow with healthful rapidity. When that is the case, that part of the body first begins to feel the effects of the stagnation which is for the time the weakest—physiologically speaking, the least able to pass the fluid along. In the case of the suicide, it is strictly, literally, and physiologically true, that for the moment the brain is the weakest point—made weak by previous intense thought, by over-exercise. But another calamity occurs just here from a double cause—there is too much blood in the brain—consequently, the busy workers of the body have not room enough to perform their accustomed part, and it is not done well; as would be the case in any department of life around us. But again, the nervous energy is made out of the blood, and if the material is impure, it is a physical necessity that the product is imperfect, is crude: hence a diseased man is fit for nothing until he gets well, and least of all for occupying the place of leaders and instructors of the people. Hence, get out of the way, all you dyspeptic editors, law-makers and teachers; you are not fit for your posts, without a puke or a purge, or a few days' bread and water diet.

There was true philosophy in the practice of a noted personage, who entered upon the consideration of all questions of great public interest by taking an emetic. He would have been a wiser man had he begun twenty-four hours sooner, to live on half allowance of food, and that of a plain substantial character.

Our main point is constantly verified in attempted suicides, by cutting the throat. By the time a quart of blood is lost, so as to relieve the brain, it becomes as clear as a bell in its activities, and the loudest call for a "doctor" comes from the patient himself—or if shame restrains, there is exhibited a very lamb-like submission to the means of restoration. Many an attempter of drowning has been known, after the shock of the plunge, to scramble for the shore with the most edifying alacrity, the shock having started the accumulated fluid from the brain, which it was oppressing so dangerously.

The general use to be made of this article, is, *If you would certainly avoid the terrible fate of a suicide, live a life of temperate eating, and of moderate bodily activities.*

And as no bodily disease comes without some friendly warning, we advise thus.—When you feel low-spirited, whether with a cause or without one, eat not an atom, drink not a drop for a dozen hours, and spend half the time at least in a brisk walk or cheerful ride to the country. You will find this a pleasant remedy, and almost infallible. And if you wish a permanent deliverance from such unwelcome visitors as the "*Blues*," eat less, exercise more.

